# Polyoxypregnane Aryl Esters Prepared from *Metaplexis japonica* (Thunb.) Makino and Their Role in Reversing Multidrug Resistance in HepG2/Dox Cells

**DOI:** 10.3390/ph18081187

**Published:** 2025-08-12

**Authors:** Yujia Guo, Huiwen Wu, Taorui Wu, Xiaoling Shen, Yingjie Hu

**Affiliations:** Science and Technology Innovation Center, Guangzhou University of Chinese Medicine, Guangzhou 510405, China; gyj13307541655@163.com (Y.G.); wuhuiwen00@yeah.net (H.W.); 13049291682@163.com (T.W.)

**Keywords:** polyoxypregnane aryl esters, *Metaplexis japonica*, multidrug resistance, P-glycoprotein, cancer cell

## Abstract

**Objective**: The development of natural and new P-gp modulators to reverse tumor multidrug resistance (MDR). **Methods**: Test compounds were prepared from the plant *Metaplexis japonica*, and their ability to reverse P-glycoprotein (P-gp)-mediated MDR was investigated in HepG2/Dox cells. Their effects on P-gp expression and function and their interaction modes with P-gp were also investigated. **Results**: Natural product 3β,12β,14β, 17β,20(*S*)-pentahydroxy-5α-pregnan-12β-*O*-(*E*)-cinnamate (**1**) and its new semisynthetic derivative 3β12β,14β,17β,20(*S*)-pentahydroxy-5α-pregnan-3β-*O*-nicotinate-12β-*O*-(E)-cinnamate (**1a**) were obtained. At non-cytotoxic concentrations of 5 or 10 μM, they significantly reversed the resistance of HepG2/Dox cells to P-gp substrate drugs doxorubicin, paclitaxel, and vinblastine, with reversal folds of 7.1, 118.5, and 198.3 (**1**), and 18.8, 335.8, and 140.0 (**1a**), respectively, at 10 μM. Cell apoptosis and expression of caspase 9 were both triggered by the combination of 10 μM of compound **1** or **1a** and 500 nM of paclitaxel (*p* < 0.001). Compound **1** or **1a** did not affect P-gp expression, but it did significantly suppress the efflux of Rhodamine 123 out of HepG2/Dox cells (*p* < 0.001). On the Caco-2 cell monolayer, **1** and **1a** were shown to be non-substrates of P-gp, with efflux ratios of 0.83 and 0.89. Molecular docking revealed their strong binding energies (−8.2 and −8.4 kcal/mol) with P-gp, and their direct binding to P-gp was confirmed by their dissociation constants (5.53 µM for **1** and 3.72 µM for **1a**), determined using surface plasmon resonance. **Conclusions**: Compounds **1** and **1a** are potential P-gp modulators; they may reverse P-gp-MDR through interacting with P-gp to interfere with substrate binding and transporting, and have the potential to improve the efficacy of paclitaxel or vinblastine drugs for combating P-gp-mediated MDR in tumor cells.

## 1. Introduction

In 2022, there were 19.98 million new cases of cancer globally, of which 24.2% were in China. Cancer has a significant impact on health, families, and society and is a major global public health issue [1]. After chemotherapy, tumors can develop resistance to not only one anticancer drug, but many others with different structures and mechanisms of action. This cross-resistance is called multidrug resistance (MDR). MDR is the main cause of tumor recurrence, metastasis, chemotherapy failure, and death in more than 90% of tumor patients. Overcoming MDR in tumor cells and improving the effectiveness of chemotherapy are key issues to be solved in the field of antitumor drug research and development [2]. MDR can develop in cancer cells in different ways, with an increase in drug efflux being a crucial factor. ATP-binding cassette transporters (ABC transporters) belong to the superfamily of transmembrane proteins, of which P-glycoprotein (P-gp), encoded by the *ABCB1* gene, is the most extensively studied. This transporter can mediate the efflux of many drugs with different structures using ATP hydrolysis to provide energy [3]. It is known that P-gp is involved in the efflux of more than 200 drugs, reducing their accumulation in cells and thus causing drug resistance [4]. MDR-reversing agents can enhance or restore the sensitivity of MDR cancer cells to chemotherapy drugs. Developing such agents or modulators is an important strategy to solve MDR in tumor cells. So far, a variety of MDR reversal agents have been developed, most of which are small-molecule compounds with different chemical structures, including early first-generation P-gp modulators such as verapamil and cyclosporin A and second-generation modulators such as dexverapamil. Third-generation P-gp inhibitors have also been discovered in the past decade, such as Zosuquidar and Tariquidar. The former has not been applied clinically due to clear toxic side effects, while the latter has entered phase II and III clinical trials [5]. Traditional Chinese medicine and natural products are important resources for discovering MDR-reversing agents [6]. Our research group previously found that the ester derivatives of tenacigenin B, a kind of polyoxypregnane (C_21_ steroid) isolated from a Chinese herbal medicine Tong-guang-san from Apocynaceae, could significantly reverse P-gp-mediated MDR in vitro and in vivo [7,8,9]. *Metaplexis japonica* (Thunb.) Makino is a plant in the same family as Tong-guang-san but contains C_21_ steroids with a steroidal skeleton slightly different from that of tenacigenin B [10,11]. In this paper, we report the results of our preliminary study on compounds derived from *M. japonica* and their MDR reversal effects.

## 2. Results and Discussion

### 2.1. Structural Identification of Compounds ***1*** and ***1a***

**Figure 1:**
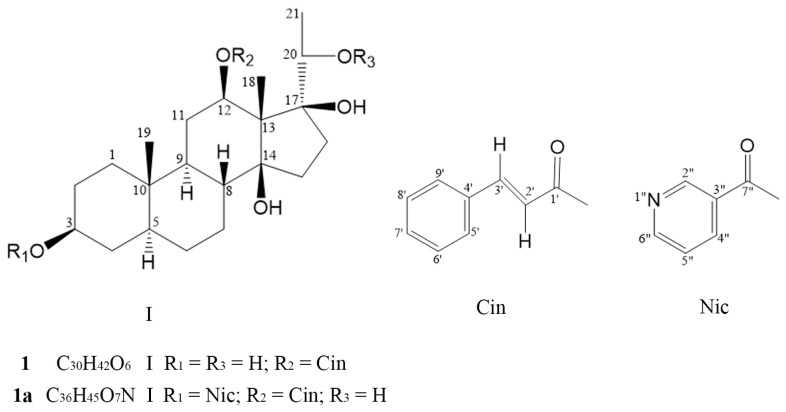
Chemical structures of compounds **1** and **1a**.

Compound **1**, white powder. UV (MeOH) λ_max_ (log ε): 205 (2.65), 217 (2.64), 280 (2.79) nm. IR (KBr) ν_max_ 3388 (broad and strong), 2930, 2859, 1780 (broad and strong), 1636, 1449, 1283, 1204, 1171, 1034, 984, 957, 770, 713 and 685 cm^−1^. High-resolution electrospray mass spectrometry (HR-ESIMS) (positive-ion mode) *m*/*z* for C_30_H_42_O_6_ (em 498.2976): 521.2860 (M + Na)^+^. ^1^H- and ^13^C-nuclear magnetic resonance (NMR) data: *δ*_H_ 7.752 (1H, d, *J* = 16.2 Hz), 7.547–7.565 (2H, m), 7.417–7.444 (3H, m), 6.446 (1H, d, *J* = 16.2 Hz) and δ_C_ 166.0 (C-1′), 146.3 (C-3′), 133.9 (C-4′), 130.8 (C-7′), 129.0 (C-6′, C-8′), 128.3 (C-5′, C-9′), and 117.3 (C-2′) showed a trans cinnamon acyl (C_9_ fragment). It conforms to the structure of pregnane with a cinnamate (C_21_ + C_9_). The δ_H_ 4.679 (1H, dd, *J* = 9.6, 4.8 Hz) in the ^1^H-NMR spectrum may be characteristic of 12α-H, with the 12β-hydroxyl group being acylated. The heteronuclear multiple bond correlation (HMBC) spectrum indicated that 12α-H is long-range correlated with the cinnamyl carbonyl (δ_C_ 166.1), proving that cinnamyl is associated with the 12β–OH group. One characteristic quadruple-peak proton signal (δ_H_ 3.641, *J* = 6.0 Hz) and one double-peak methyl signal (δ_H_ 1.119, *J* = 6.0 HZ, 21-CH_3_) in the ^1^H-NMR spectrum indicate the presence of a 20-OH and no proton at the C-17 position coupling with 20-H. The hepta-peak at δ_H_ 3.623 agreed with the characteristics of the 3α -hydrogen signal. Thus, the structure of compound **1** was identified as 3β,12β,14β,17β,20(*S*)-pentahydroxy-5α-pregnan-12β-*O*-(*E*)-cinnamate (Figure 1). Compound **1** was earlier reported to be the saponified product of tomentodin [12β-*O*-(*E*)-cinnamoyl-20(*S*)-*O*-acetyl-tomentogenin] [12] and has been isolated and identified as a natural product from a plant for the first time. The assignment of ^1^H- and ^13^C-NMR data for **1** is shown in Table 1.

Compound **1a**, white amorphous powder. HR-ESIMS (positive-ion mode) *m*/*z* for C_36_H_45_O_7_N (em 603.3189): 604.3271 (M + H)^+^ and 36 carbon signals in the ^13^C-NMR spectrum indicated that **1a** is a mono-nicotinate of **1** (C_30_H_41_O_6_ + C_6_H_4_ON) [12]. The ^1^H-NMR spectra showed three methyl signals, δ_H_ 1.341 (s), 1.122 (d, 6.4 Hz), and 0.908 (s), of steroidal aglycons in the high-field region. Among them, the double-peak methyl signal at of δ_H_ 1.122 should be coupled with a proton at δ_H_ 3.647 (d, 6.4 Hz), suggesting that the latter should be 20-hydrogen and the 17-carbon is connected with a hydroxyl group, conforming to the characteristics of compound **1**. The NMR spectrum of **1a** showed characteristics of cinnamyl, such as δ_H_ 7.755 and 6.444 (each 1H, dd, *J* = 15.6, 2.0 Hz, 3′-H and 2′-H), and the ^1^H-^1^H correlated spectroscopy (^1^H-^1^H COSY) spectrum shows mutual coupling between the two protons. *J* = 2.0 Hz indicates long-range coupling protons from 5′-H and 9′-H on the benzene ring. Features of a nicotinate moiety also appeared, such as δ_H_ 9.202 (1H, s, 2″-H), 8.756 (1H, d, *J* = 4.8 Hz, 6″-H), 8.293 (1H, dd, *J* = 8.0, 4.8 Hz, 5″-H), and 7.42–7.37 (4H, m, 4″-H, 6″-H, as well as 7′-H and 8′-H). A proton signal at δ_H_ 7.390 identified in COSY and HMBC spectra could be ascribed to 4′-H, as it was coupled with both the signal of 5′-H (δ_H_ 8.756) and the δ_C_ signals including 150.8 (C-2″), 126.7 (C-3″), 123.3 (C-4″), 137.1 (C-5″), 153.2 (C-6″), and 164.7 (C-7″). Both the 3β-OH and 12β-OH groups are esterified, and the electronegative effect of the ester groups caused the two characteristic protons at δ 4.993 (hept, 4.8 Hz, 3α-H) and 4.692 (dd, 11.6, 4.4 Hz, 12α-H) to shift to the low field. Since the proton signal at δ 4.692 is consistent with that of compound **1**, the hepta-peak signal can be assigned to 3α-H. We found that the 21-methyl protons at δ 1.122 not only correlated to the 20-H (doublet at δ 3.647; ^1^H–^1^H COSY spectrum) but also to the carbon signals of δ_C_ 71.4 and 88.1 (HMBC spectrum), indicating that these two carbons should be assigned as C-20 and C-17, respectively. Therefore, the structure of compound **1a** was established as 3β,12β,14β,17β,20(*S*)-pentahydroxy-5α-pregnan-3β-*O*-nicotinate-12β-*O*-(*E*)-cinnamate (Figure 1). The assignment of ^1^H- and ^13^C-NMR data for **1a** is shown in Table 1.

### 2.2. Compounds ***1*** and ***1a*** Significantly Reversed P-gp-Mediated MDR in HepG2/Dox Cells

It was found in a previous study that aromatic acyl-substituted 7,8-pyranocoumarins exhibited higher P-gp-mediated MDR-reversing ability when compared with fatty acyl substituted 7,8-pyranocoumarins [13]; di-esterified tenacigenin B could reverse P-gp-mediated MDR in cancer both in vitro and in vivo, whereas tenacigenin B could not [7,8]. Nicotinic acid (also called Vitamin B_3_), exhibiting good bioactivity and safety, has a wide range of uses. Nicotinic esterification can improve the bioactivity of natural products [14,15]. Based on the above considerations, we prepared compound **1a**, the nicotinate of compound **1**, and investigated the effects of both on P-gp-mediated MDR in HepG2/Dox cells. They were investigated because a variety of clinically used antitumor drugs are P-gp substrates and recurrent tumors usually express high levels of P-gp.

**Figure 2:**
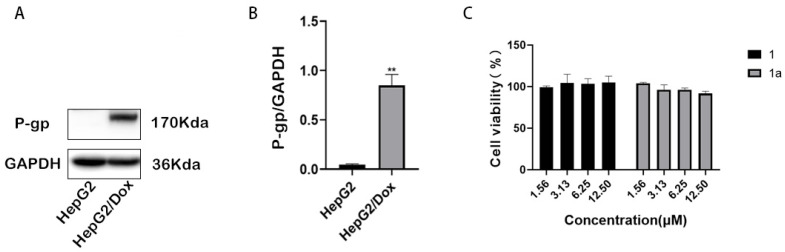
P-gp expression in HepG2 and HepG2/Dox cells. (**A**) Detection of P-gp by Western blotting. (**B**) Quantitative analysis of the protein bands. (**C**) Viability of HepG2/Dox cells after a 48 h treatment with compound **1** or **1a**. Compared with HepG2, ** *p* < 0.01.

The results after 48 h of treatment are presented in Figure 2 and Table 2. HepG2/Dox cells expressed a much higher level of P-gp than their parental HepG2 cells (Figure 2A,B), and were highly resistant to doxorubicin, paclitaxel, and vinblastine (all are P-gp substrates), with the resistance folds (F_res_) as high as 35.5, 130, and 140, respectively. The P-gp modulator verapamil (VRP) effectively reversed the resistance of cells to the above three drugs, as expected. In this study, compounds **1** and **1a**, at their non-toxic concentration of 5 μM or 10 μM (Figure 2C), reduced the resistance of HepG2/Dox cells to all three drugs in a concentration-dependent manner, in the same way as verapamil. At 10 μM, they even completely reversed the resistance of cells to paclitaxel and vinblastine; compound **1** increased the sensitivity of HepG2/Dox cells to paclitaxel and vinblastine by 118.5 and 198.3 times, and compound **1a** increased their sensitivity by 335.8 and 140 times (see Graphical Abstract), showing a stronger ability than VRP. Compounds **1** and **1a** were non-cytotoxic at 5 or 10 μM (Figure 2C; Supplementary Materials Figure S1). Considering that 10 μM, or even 5 μM, is not a low concentration for an in vitro experiment, it can be stated that these two compounds are much safer than cytotoxic anticancer drugs, and thereby have a safety advantage when used as chemosensitizers in combination with anticancer drugs.

### 2.3. Compounds ***1*** and ***1a*** Significantly Enhanced Paclitaxel-Induced Intrinsic Apoptosis in HepG2/Dox Cells

**Figure 3:**
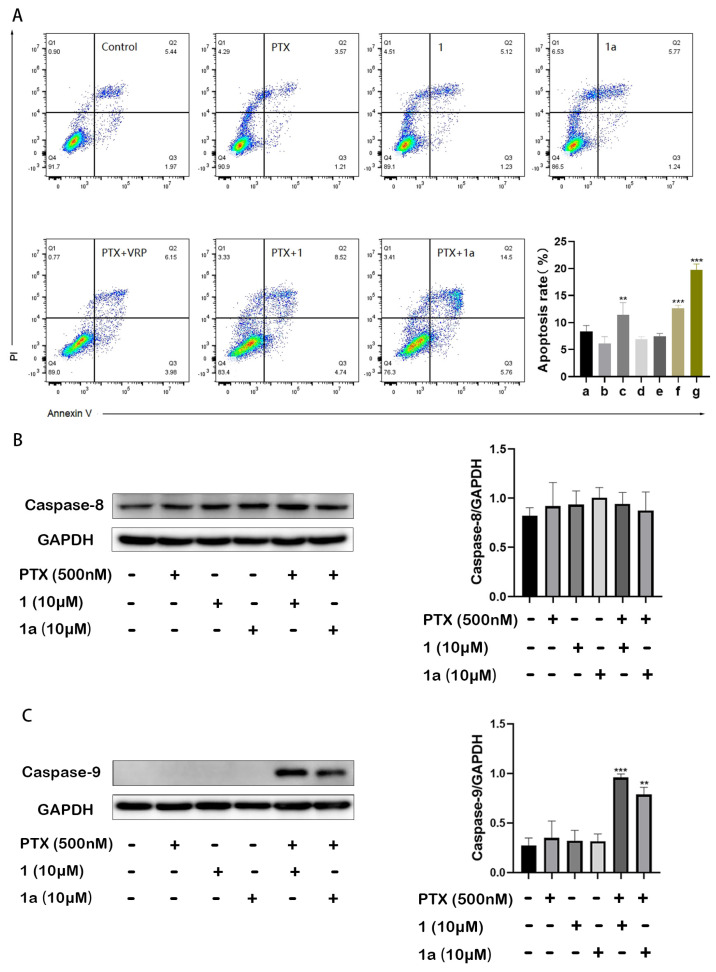
Compounds **1** and **1a** significantly enhanced paclitaxel-induced apoptosis in HepG2/Dox cells. Cell apoptosis was recorded using flow cytometry (**A**), cellular expression levels of caspase 8 (**B**) and caspase 9 (**C**) were analyzed via Western blotting. a. Control; b. PTX (500 nM); c. PTX (500 nM) + VRP (10 μM); d. compound **1** (10 μM); e. compound **1a** (10 μM); f. PTX (500 nM) + compound **1** (10 μM); g. PTX (500 nM) + compound **1a** (10 μM). Compared with PTX alone, ** *p* < 0.01 and *** *p* < 0.001.

We further investigated the effect of **1** and **1a** on paclitaxel-induced apoptosis in HepG2/Dox cells. As shown in Figure 3A, treatment with 500 nM paclitaxel (PTX) alone for 24 h did not induce cell apoptosis due to drug resistance, but the apoptosis rates of HepG2/Dox cells were significantly increased when the drug was used in combination with a non-cytotoxic concentration (10 μM) of **1**, **1a**, or VRP (*** *p* < 0.001 for **1** and **1a**; ** *p* < 0.01 for VRP). The results further confirmed the ability of **1** and **1a** to reverse P-gp-mediated MDR in tumor cells.

Upregulated protein expressions of caspase 8 and caspase 9 can be observed in paclitaxel-induced apoptosis in cancer cells. The enhanced expression of the former indicates that apoptosis occurs through an extrinsic (death receptor-related) apoptotic pathway, while that of the latter suggests that apoptosis is achieved through an intrinsic (mitochondrial) apoptotic pathway [16,17]. In this study, we observed the expressions of caspase 8 and caspase 9 in hepatocellular cells and the effects of the drug on protein expression. The results showed that the multidrug-resistant HepG2/Dox cells already have highly expressed caspase 8 but low expression of caspase 9. The ineffective concentration (0.5 μM) of paclitaxel and the non-cytotoxic concentration (10 μM) of compound **1** or **1a** alone did not change the expressions of either caspase 8 or caspase 9. However, when compound **1** or **1a** was combined with paclitaxel at the mentioned concentration, the already high expression of caspase 8 did not change, while that of caspase 9 was significantly upregulated. This result demonstrates that compounds **1** and **1a** exert synergistic anticancer effects by enhancing the extrinsic (mitochondrial) apoptosis induced by paclitaxel.

### 2.4. Compounds ***1*** and ***1a*** Did Not Affect the Expression but Inhibited the Function of P-gp in HepG2/Dox Cells

**Figure 4:**
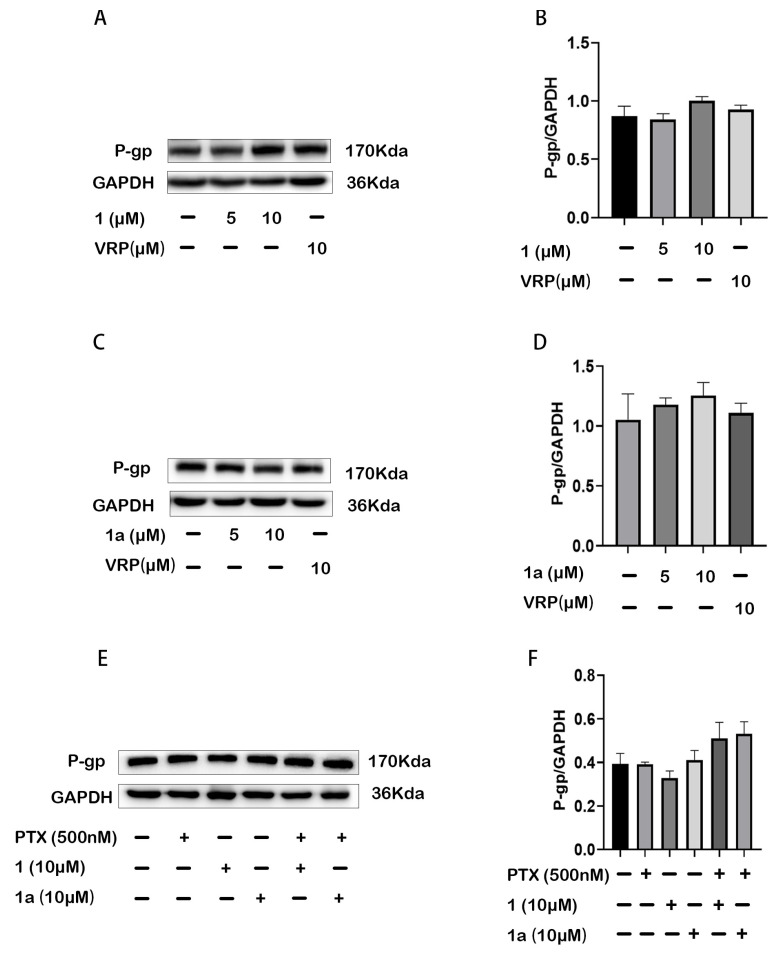
Compound **1** or **1a** did not affect P-gp expression in HepG2/Dox cells. (**A**,**C**,**E**) Detection of P-gp expression by Western blotting; (**B**,**D**,**F**) Quantitative analysis of the protein bands.

P-gp is an energy-dependent membrane transporter; it actively pumps its substrates out of the cell. Thus, the reversal of P-gp-MDR might be achieved by either suppressing the expression of P-gp or inhibiting its transport function. In this study, the effects of compound **1** or **1a** on the expression and pump function of P-gp in HepG2/Dox cells were investigated. Our results showed that compounds **1** and **1a** did not influence the expression level of cellular P-gp, whether they were used alone or in combination with paclitaxel, showing activity similar to VRP (Figure 4). Therefore, the results indicate that the suppression of P-gp expression was not the mechanism by which compounds **1** and **1a** reversed P-gp MDR.

**Figure 5:**
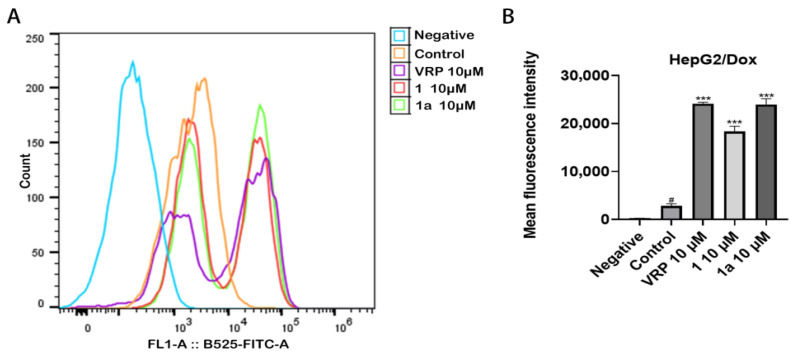
Compounds **1** and **1a** significantly inhibited the efflux of Rh-123 from HepG2/Dox cells. (**A**) Detection of cellular fluorescence using flow cytometry. (**B**) Quantitative analysis of cellular fluorescence. Negative group: cells without Rh-123; control group: cells incubated with Rh-123 alone. # *p* < 0.05, compared with negative group; *** *p* < 0.001, compared with PTX (500 nM) group.

Following this, we used Rhodamine 123 (Rh-123), a fluorescent P-gp substrate, as an indicator to investigate the effect of compounds **1** and **1a** on P-gp-mediated substrate transport, and the results are shown in Figure 5. Compared to the fluorescence intensity of Rh-123 in control cells that were incubated with Rh-123 alone, that in cells incubated with Rh-123 in the presence of compound **1**, **1a**, or VRP was significantly increased (*** *p* < 0.001). The inhibited efflux of Rh-123 by compounds **1** and **1a** suggests that these two compounds may reverse the resistance of HepG2/Dox cells to anticancer drugs that are P-gp substrates by inhibiting the transport function of P-gp.

### 2.5. Compounds ***1*** and ***1a*** Acted as Non-Substrates of P-gp in Caco-2 Cell Monolayer

The above results reveal that compounds **1** and **1a** reverse MDR through regulating P-gp function; however, it is not clear whether they interact with P-gp as a substrate or non-substrate. Caco-2 cell monolayers with highly expressed P-gp are commonly used to mimic the barrier function and drug transport process of human small intestinal epithelium and evaluate the intestinal absorption, transport, excretion, or uptake effects of drugs [18]. In this study, whether compounds **1** and **1a** are P-gp substrates or not was investigated by employing the Caco-2 cell monolayer model, using digoxin (P-gp substrate) as the positive control and efflux ratio as the indicator. Our results are presented in Table 3. In this experiment, digoxin exhibited an efflux ratio (ER) as high as 181, indicating mature Caco-2 monolayers with stably expressed P-gp; compounds **1** and **1a** showed ER values of less than 2 (0.83 for compound **1** and 0.89 for compound **1a**), indicating that they are not P-gp substrates and could not be transported. The results suggested that compounds **1** and **1a** interact with P-gp in non-substrate modes. In addition, the measured apparent permeability coefficients (Papp) from AP to BL are 9.28 × 10^−6^ cm/s for compound **1** and 0.27 × 10^−6^ cm·s^−1^ for compound **1a**, inferring high permeability of compound **1** and low permeability of compound **1a**. Meanwhile, a solution recovery of less than 55% for both suggested that the two compounds may have obvious specific adsorption or cellular metabolism. Our data also suggest that compounds **1** and **1a** may have the potential to enhance the oral bioavailability of P-gp substrate drugs paclitaxel and vinblastine, which is promising for the development of oral preparations.

### 2.6. Compounds ***1*** and ***1a*** Interact with P-gp with High Affinity but at Different Sites

**Figure 6:**
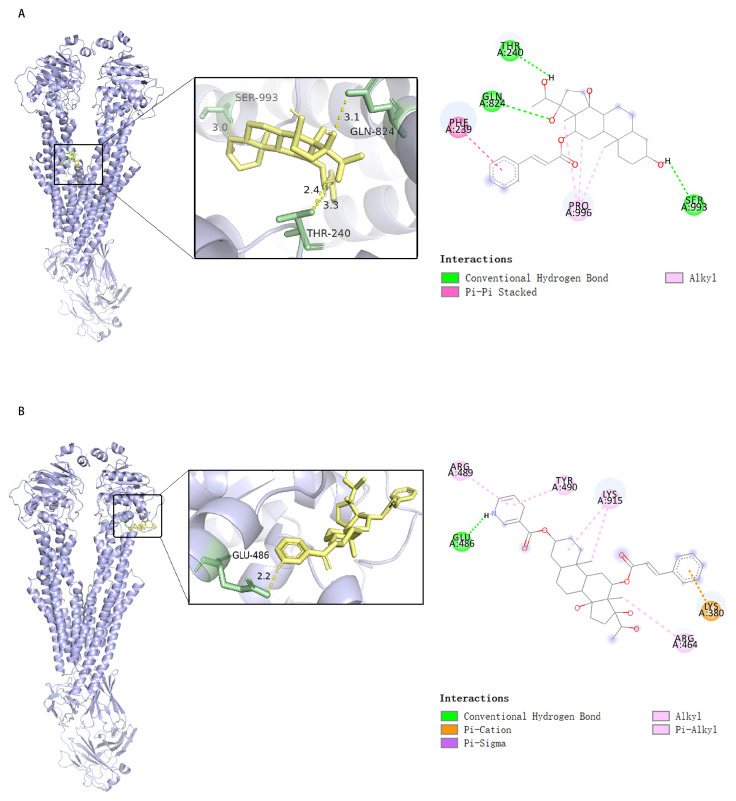
Visualization diagrams of docking of P-gp to **1** and **1a** (**left**: 3-D diagram; **right**: 2-D diagram). (**A**) P-gp docking to compound **1**; (**B**) P-gp docking to compound **1a**.

To further reveal the interaction modes of compounds **1** and **1a** with P-gp, their molecular docking to P-gp was modeled [19] and the results are presented in Figure 6. The results show that compound **1** may interact with P-gp at the GLN-824, THR-240, and SER-993 residues via hydrogen bonding, at the PHE-239 residue via Pi-Pi stacking, and at the PRO-996 residue via hydrophobic interactions, with a calculated binding energy of −8.2 kcal/mol (Figure 6A). Compound **1a** may interact with P-gp at the GLU-486 residue via hydrogen bonding, at the ARG-489, TYR-490, LYS-915, and ARG-464 residues via hydrophobic interactions, and at the LYS-380 residue via Pi–cation interactions, with a calculated binding energy of −8.4 kcal/mol (Figure 6B). The results suggest that compounds **1** and **1a** may interact with P-gp to form stable complexes, interfere with substrate binding, and thus inhibit P-gp-mediated substrate efflux.

**Figure 7:**
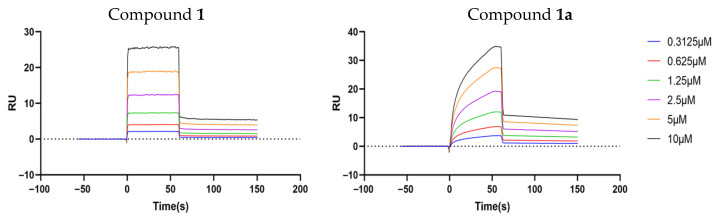
Response (RU)–concentration curves from SPR analyses of the binding of compound **1** or **1a** to full-length human ABCB1.

Validation of the above results was performed through surface plasmon resonance (SPR) analyses [20,21] on the Biacore 8K system. The measured equilibrium dissociation constants (K_D_), which represent the degree of affinity of the analytes to their target (P-gp; human ABCB1), were as low as 5.53 μM for compound **1** and 3.72 μM for compound **1a**, confirming the direct binding between P-glycoprotein and compound **1** or **1a**, while the high association rate constant (Ka) (>10^4^ M^−1^s^−1^) and dissociation rate constant (Kd) (>10^−1^ s^−1^) indicated a fast-binding and fast-dissociating interaction mode (Table 4). In addition, the response–concentration curve of compound **1** supports a hydrogen bond-based interaction with P-gp, while that of compound **1a** shows that, despite hydrogen binding, hydrophobic and other interactions also contribute to the binding of compound **1a** and P-gp (Figure 7).

The calcium channel inhibitor verapamil is a substrate of P-gp that increases intracellular accumulation of anticancer agents that are P-gp substrates by functioning as a competitive inhibitor [22]. It is now considered a typical representative of the first-generation of P-gp inhibitors. Unlike verapamil, plant-originated steroids **1** and **1a** are not P-gp substrates. The effects of steroid drugs on overcoming P-gp-mediated MDR in cancer cells have been revealed. Progesterone (pregnan-4-3,20-dione), with a pregnane (C_21_ steroid) structure that resembles that of compounds **1** and **1a**, interacts with P-gp to inhibit the binding of P-gp substrate drugs, thus increasing the drug sensitivity of cancer cells with overexpressed P-gp [23]. Estrogen 17β-estradiol downregulates P-gp expression in MDR1-transduced, estrogen receptor-positive human breast cancer cells [24]. As a sex hormone, the structure of estrogen is characterized by arylated ring A, a 3-phenolic hydroxy group, 19-demethyl, and an absence of the C-17 side chain in its steroidal molecule, and thus significantly differs from the structures of compounds **1** and **1a**. The following are the mechanisms by which natural polyoxypregnane ester compounds reverse P-gp-mediated MDR: (1) They inhibit the protein expression of P-gp. Wu et al. showed that the compound MT2 (11α-*O*-2-methylbutanoyl-12β-*O*-tigloyltenacigenin B) from Marsdenia tenacissima suppressed P-gp and MRP2 expression in vitro and in vivo to re-sensitize the effect of paclitaxel in the treatment of MDR cervical cancer [9]. Yuan et al. [25] demonstrated that asclepiasterol, identified from Asclepias curassavica, reverses MDR in cancer by downregulating P-gp expression in multidrug-resistant breast cancer cells and hepatoma cells. (2) They suppress the function of P-gp by interfering in its binding with substrate drugs, such as the series of tenacigenin B derivatives isolated from Marsdenia tenacissima by To et al. [26]. In addition to the above routes, several C-11- and C-12-substitutive ester derivatives of polyoxypregnane isolated from plants have been found to inhibit liver microsomal drug-metabolizing enzymes, especially CYP3A4, and enhance the in vivo efficacy of paclitaxel on cervical tumor xenografts, indicating that esterification to increase hydrophobicity to polyoxypregnane is essential for inhibition of both the expression and function of P-gp and CYP3A [7,8,9,27]. We proved that compounds **1** and **1a** are MDR reversal agents that directly bind to P-gp in non-substrate ways; thus, they work through interfering with the transport function of P-gp, rather than through inhibiting the protein expression of P-gp. However, further research is required to determine whether they can work through increasing cellular membrane rigidity to impair the efflux function of P-gp, reducing P-gp-ATPase activity, or inhibiting metabolic enzymes.

## 3. Materials and Methods

### 3.1. Materials and Reagents

The plant was collected in Jianshui County, Yunnan Province, China, and identified by Professor Hua Peng of Kunming Institute of Botany, Chinese Academy of Sciences as *Metaplexis japonica* (Thunb.) Makino, a plant of Apocynaceae. The specimen (#LM 2021-0704) was preserved in the phytochemistry laboratory of the Science and Technology Innovation Center, Guangzhou University of Chinese Medicine. The test compounds were prepared by the authors, and the purity of the compounds were detected via HPLC to be ≥98.0%. The organic solvents and chemical reagents used in this study were AR-grade (China). Paclitaxel, doxorubicin, and vinblastine were purchased from Sigma (St Louis, MO, USA). Verapamil was obtained from MP Biomedicals (Irvine, CA, USA). RPMI 1640 and trypsin were purchased from Biological Industries (Cromwell, CT, USA); fetal bovine serum (FBS) from Gibco (Grand Island, NY, USA); Cell Counting Kit 8 (CCK-8) from GLPBIO (Montclair, CA, USA); P-gp antibody from Hangzhou Hua’an Biotechnology (Hangzhou, China); GAPDH antibody from Affinity Corporation (San Francisco, CA, USA); rabbit secondary antibody from SAB Company (Fairfield, NJ, USA); and BCA Protein Concentration Determination Kit, Rhodamine 123, and Annexin-V/PI Double Staining Kit from Shanghai Beyotime Technology (Shanghai, China).

### 3.2. Equipment

The equipment used in the experiment comprised AVANCE NEO Ascend 600 and AVANCE NEO 400 nuclear magnetic resonance (NMR) spectrometers (Bruker, Berlin, Germany), a 1290 Infinity IO-6546 ultra-performance liquid chromatography–quadrupole time-of-flight mass spectrometer (UPLC-Q-TOF-MS) (Agilent, Santa Clara, CA, USA), a Nicolet iS20 infrared spectrometer (Thermo Scientific, Waltham, MA, USA), an LC1260 high-performance liquid chromatograph (HPLC) (Agilent, Santa Clara, CA, USA), an HF90 carbon dioxide incubator for cell culture (Shanghai Heal Force, Shanghai, China), a 041BR159108 electrophoresis apparatus (041BR159108 Type), a ChemiDoc chemiluminescence scanner (BIO-RAD, Hercules, CA, USA), and a CytoFLEX flow cytometer (Beckman Coulter, Indianapolis, IN, USA).

### 3.3. Synthesis of Compounds ***1*** and ***1a***

The dried and powdered plant sample of *M. japonica* (3.5 kg) was extracted with 90% ethanol (1:8, *w*/*v*) under reflux three times (2 h each time). Then, the combined extraction solution was distilled in vacuum to remove ethanol. The aqueous residue was extracted with ethyl acetate five times, and the combined ethyl acetate solution was distilled and evaporated under vacuum to prepare the dried residue (208.0 g). Half of the dried residue was dissolved in 95% ethanol (1000 mL), and then 1000 mL of 0.20 mol/L sulfuric acid water solution was added. After 1 h of reaction under reflux, the solution was cooled to 40 °C, adjusted to pH 7 with 30% sodium hydroxide (*w*/*v*), distilled in vacuum to remove the ethanol, and then extracted with ethyl acetate five times. The combined acetate solution was distilled and evaporated in vacuum to obtain a dried residue (92.1 g); this obtained residue was named total aglycone, which gave a positive color reaction to Liebermann–Burchard’s reagent [28]. Sixty grams of the total aglycon was added to a chromatographic column with silica gel (200–300 mesh) as stationary phase and eluted with petrol ether (b.p. 60–90 °C)–acetone (95:5~70:30, *v*/*v*), at 200 mL per fraction. Components in the fraction were monitored using thin-layer chromatography (TLC) and the color-developing reagent contained 3% (*w*/*v*) vanillin and 6% perchloric acid (*w*/*v*) in 50% ethanol (*v*/*v*). From the fraction No. 17~19, which was eluted using petrol ether–acetone (80:20), a single compound **1** (3.40 g) was obtained with a fairly high yield.

In a 25 mL flask, compound **1** (50 mg, 0.10 mmol), 4-dimethylaminopyridine (25 mg, 0.30 mmol), nicotinoyl chloride (35 mg, 0.25 mmol), and anhydrous pyridine (0.5 mL) were added, mixed well, and stirred magnetically at RT (27 °C) for 3 h. When compound **1** could no longer be detected via TLC, 2 mL ethanol was added, the mixture was left to stand at RT for 0.5 h, 1 mL water was added, and then the mixture was dried in vacuum on a water bath. Compound **1a** (44.5 mg) was isolated and purified using silica gel column chromatography (CC) with a yield of 74%. The TLC and CC solvents were dichloromethane–methanol (98:2, *v*/*v*).

### 3.4. Evaluation of MDR Reversal Effects of Compound ***1*** and ***1a***

#### 3.4.1. Cell Line and Cell Culture

Human hepatic carcinoma cell line HepG2 was purchased from National Collection of Authentic Cell Cultures (Shanghai, China). Doxorubicin-induced P-gp overexpression HepG2/Dox subline was a gift from Prof. Fong (retired) of City University of Hong Kong [7,13]. Cells were maintained in RPMI 1640 containing 10% FBS and 100 U Penicillin-Streptomycin, at 37 °C, in a 5% CO_2_ incubator with saturated humidity. In the HepG2/Dox cell medium, 1.2 μM doxorubicin was added to maintain the drug tolerance of the cell; the cells were required to grow in doxorubicin-free medium for at least 6 days before use in the experiments.

#### 3.4.2. Detection of Cell Viability via Cell Counting Kit 8

In 96-well plates, 5000 cells in 100 µL medium were seeded to each well. After 24 h of incubation, cells in 100 µL fresh medium were treated with different concentrations of paclitaxel, doxorubicin, or vinblastine for 48 h, with or without a non-toxic concentration (5 μM or 10 μM) of the test compound (**1** or **1a**), or verapamil. After that, cell viability was assessed using CCK-8 by following the manufacturer’s instructions. The inhibitory effect of an anticancer agent on cell proliferation was analyzed and expressed as median inhibitory concentration (IC_50_). The ability of 1 and 1a to reverse drug resistance was evaluated using the fold decrease in IC_50_ of the anticancer agent achieved.

#### 3.4.3. Observation of Apoptosis via Annexin V/PI Staining

In the 6-well plates, 4 × 10^5^ HepG2/Dox cells in 2 mL medium were seeded and incubated for 24 h. Cells were then incubated for another 24 h in 2 mL fresh medium containing the test samples. The medium and cells (digested by EDTA-free trypsin) were collected and centrifuged (1000 rpm, 5 min). The cells were then suspended in PBS and stained using the Annexin V/PI kit by following the procedures provided with the kit, and the apoptosis rate of cells with different treatments was analyzed using flow cytometry.

### 3.5. Study on the MDR-Reversing Mechanism of Test Compounds

#### 3.5.1. Detection of Cellular Protein Expression via Western Blotting

In 100 mm culture dishes, 1 × 10^6^ cells in 10 mL medium were seeded and incubated for 24 h, and then incubated with the test sample in 10 mL fresh medium for another 24 h. Total proteins were extracted using RIPA cell lysis buffer containing PMSF while protein content was determined using the BCA kit. An equal amount of the protein sample was subjected to SDS-PAGE for separation and transferred to PVDF membranes. The membranes were blocked in 5% defat milk at RT for 1 h, and then incubated with the primary antibody of the protein of interest (P-gp, caspase 8 or caspase 9) or GAPDH (internal standard) at 4 °C overnight, and then with the secondary antibody at RT for 1 h. Protein bands were detected under the chemiluminescence scanner, while Image J was employed to analyze the gray value of the protein bands.

#### 3.5.2. Rhodamine 123 Efflux Assay for Detection of P-gp Function

In the cell culture incubator, 5 × 10^5^ HepG2/Dox cells suspended in 1 mL medium were incubated with 5 μM Rh-123 in the presence or absence of 10 μM of compounds **1**, **1a**, or VRP for 40 min, and then cooled to 4 °C on ice. After removal of the medium via centrifugation, the cells were washed with ice-cold PBS twice, resuspended in 300 μL ice-cold PBS, and transferred into flow cytometer tubes. Cellular fluorescence intensity was analyzed immediately using flow cytometry.

#### 3.5.3. Bidirectional Transport Assay on Caco-2 Cell Monolayers

Caco-2 cells (ATCC) in MEM containing 10% FBS and 1% NEFA were seeded onto 0.4 μm pore polycarbonate (PC) membranes in 96-well Corning Insert plates (3.5 × 10^4^ cells/cm^2^). The plates were placed in a CO_2_ incubator for 21~28 d to let the cells form confluent cell monolayers, with medium refreshed every 4~5 days. HBSS with 10.0 mM HEPES at pH 7.40 ± 0.05 was used as the transport buffer in the transport assay. For A→B transport, 75 μL buffer containing 2 μM compound **1, 1a**, or 10 μM digoxin was added into the apical chamber, and 250 μL buffer was added into the basolateral chamber; for B→A transport, 250 μL buffer containing 2 μM compound **1**, **1a**, or 10 μM digoxin was added into the basolateral chamber, and 75 μL buffer was added into the apical chambers. Compounds **1** and **1a** were added in triplicate while digoxin was added in duplicate. The plates were placed in the CO_2_ incubator for 2 h. After addition of the stop solution, the plates were centrifuged at 3220× *g* for 20 min. Concentrations of compound **1**, **1a**, and digoxin in all samples were semi-quantitatively determined using LC-MS/MS methodologies, using the analyte/internal standard area ratio (Supplementary Materials). After the transport assay, the lucifer yellow rejection assay was applied to determine the integrity of the Caco-2 cell monolayer.

Apparent permeability coefficient (Papp), efflux ratio (ER), and percentage recovery (solution recovery) of the test compounds were calculated with the following equations:

Papp (cm/s) = (dCr/dt) × Vr/(A × C_0_)

Efflux Ratio (ER) = Papp (B→A)/Papp (A→B)

Solution Recovery % = 100 × [(Vr × Cr) + (Vd × Cd)]/(Vd × C_0_)

where dCr/dt is the cumulative concentration of a compound in the receiver chamber as a function of time; Vr is the solution volume in the receiver chamber (0.0750 mL on the apical side, 0.250 mL on the basolateral side); A is the surface area for the transport (0.143 cm^2^ for the area of the monolayer); C_0_ is the initial concentration in the donor chamber; Vd is the volume in the donor chambers (0.0750 mL on the apical side, 0.250 mL on the basolateral side); and Cd and Cr are the final concentrations of the transport compound in the donor and receiver chambers, respectively.

### 3.6. Molecular Docking

The three-dimensional crystalline structure of P-gp (PBD code: 6QEX) was downloaded from the PBD database (https://www.rcsb.org/, accessed on 5 August 2025) and exported as a PDB file. After adding hydrogen manually via Autodocktools (1.5.7) and selecting “Receptor” (Micromolecule), the file was exported in PDBQT format. The two-dimensional structures of compounds **1** and **1a** were drawn using Chemdraw software (22.0.0), and the structures were optimized using Chem3D (22.0.0), Openbable (3.1.1), and PyMol software (3.0). After manually removing the water molecule and adding hydrogen via Autodocktools, and selecting “Ligand”, the file was exported in PBDQT format. The PDBQT files of “Ligand” and “Receptor” were imported into AutoDock Vina software (1.1.2) for docking [19], and the receptor–ligand binding mode with the lowest binding energy was recorded. The docking results and binding modes were visualized with the help of PyMOL and Discovery studio (2019 edition).

### 3.7. Surface Plasmon Resonance

The human recombinant ABCB1 full-length protein (HEK293, Flag) (MedChemExpress, Monmouth Junction, NJ, USA; #HY-P703175) was coupled onto a CM5 sensor chip (Cytiva, Marlborough, MA, USA; #BR-1005-30) via carboxyl groups on the dextran. After incubation, a series of concentrations of compound **1** or **1a** flowed through the protein–CM5 system. Binding was tested and analyzed using a Biacore K8 instrument (GE Healthcare, Chicago, IL, USA) and Biacore Insight evaluation software (https://www.cytivalifesciences.com/en/us, accessed on 5 August 2025) (Cytiva, Marlborough, MA, USA).

### 3.8. Statistical Analysis

Statistical analyses were performed using GraphPad Prism 8.0.2 software. Data were expressed as mean ± SD of three independent experiments. Differences between groups were analyzed via one-way ANOVA, and *p* < 0.05 was considered statistically significant.

## 4. Conclusions

In the present paper, the structures of a C_21_ steroidal aglycon isolated from total aglycones of *Metaplexis japonica* and its semisynthetic nicotinate were identified as 3β, 12β, 14β, 17β, 20(*S*)-pentahydroxy-5α-pregnan-12β-*O*-(*E*)-cinnamate (**1**) and 3β, 12β, 14β, 17β, 20(*S*)-pentahydroxy-5α-pregnan-3β-*O*-nicotinate-12β-*O*-(*E*)-cinnamate (**1a**) based on their spectroscopic data. Compound **1a** is a new compound. Our research also revealed remarkable multidrug resistance-reversing activity for compounds **1** and **1a** in P-gp-overexpressing hepatoma cells. It is interesting that compounds **1** and **1a**, at their non-cytotoxic concentrations, completely reversed the resistance of HepG2/Dox cells to paclitaxel and vinblastine and significantly enhanced the intrinsic cell apoptosis induced by paclitaxel through directly binding to the P-gp protein, inhibiting its function in the non-substrate mode. This shows the potential of these compounds as adjuvants to improve the effect of paclitaxel and vinblastine drugs in the treatment of P-gp-mediated multidrug-resistant cancer.

## Figures and Tables

**Table 1:** ^1^H-NMR and ^13^C-NMR data attribution for compounds **1** and **1a.**

C	1		1a		
	*δ*_H_, (*J* in Hz)	*δ*_C_	*δ*_H_, (*J* in Hz)	*δ*_C_	Correlation
1		37.8, CH_2_		36.7, CH_2_	
2		31.7, CH_2_	1.50, 1.53	27.3, CH_2_	
3	3.607 (hept, 3α)	71.0, CH	4.993 (hept, 4.8, 3α)	74.5, CH	COSY: 1.341 (18-H), 1.40, 1.53 (2-H), 1.95 (9-H)
4		36.9, CH_2_	1.57	33.8, CH_2_	
5		44.5, CH		44.4, CH	
6		28.2, CH_2_		28.2, CH_2_	
7		26.6, CH_2_		26.6, CH_2_	
8		39.3, CH		39.3, CH	
9		45.4, CH	1.95	45.3, CH	
10	/	35.5, C	/	35.6, C	
11		27.0, CH_2_	1.38, 1.40, 1.57	27.0, CH_2_	
12	4.665 (dd, 11.4, 4.2, 12α)	74.6, CH	4.692 (dd, 11.2, 4.8, 12α)	74.7, CH	COSY: 1.50, 1.53, 1.57, 1.95
13	/	55.1, C		55.1, C	HMBC: 166.1 (C-1′)
14	/	87.3, C		87.2, C	
15		30.9, CH_2_		30.9, CH_2_	
16		31.2, CH_2_		31.8, CH_2_	
17	/	88.1, C		88.1, C	
18	1.320 (s)	12.2, CH_3_	1.341 (s)	12.2, CH_3_	HMBC: 74.7 (C-12), 55.1 (C-13), 87.2 (C-14)
19	0.825 (s)	9.7, CH_3_	0.908 (s)	9.7, CH_3_	HMBC: 35.6 (C-10), δ_C_ 44.4 (C-5)
20	3.623 (q, 7.2)	71.4, CH	3.647 (q, 6.0)	71.4, CH	
21	1.111 (d, 7.2)	17.2, CH_3_	1.122 (d, 6.4)	17.2, CH_3_	COSY: 3.647 (20-H); HMBC: 71.4 (C-20), 88.1 (C-17)
1′	/	166.1, C		166.1, C	
2′	6.445 (d, 17.6)	117.2, CH	6.444 (dd, 16.2, 2.0)	117.2, CH	HMBC: 134.0 (C-4′), 166.1 (C-1′)
3′	7.745 (d, 17.6)	146.3, CH	7.755 (dd, 16.2, 2.0)	146.3, CH	HMBC: 128.3 (C-5′, C-9′), 134.0 (C-4′), 134.0 (C-4′), 117.2 (C-2′)
4′	/	133.9, C		134.0, C	
5′	7.53~7.56 (2H, m, 5′, 9′)	128.4, CH	7.54~7.56 (2H, m, 5′, 9′)	128.3, CH	
6′	7.39~7.44 (3H, m)	129.0, CH	7.41~7.37 (4H, m, 4″-H, 6′-H, 7′-H, 8′-H)	129.0, CH	HMBC: 128.3 (C-5′, C-9′)
7′	7.39~7.44 (3H, m)	130.8, CH	7.41~7.37 (4H, m, 4″-H, 6′-H, 7′-H, 8′-H)	130.8, CH	
8′	7.39~7.44 (3H, m)	129.0, CH	7.37~7.41 (4H, m, 4″-H, 6′-H, 7′-H, 8′-H)	129.0, CH	
9′	7.53~7.56 (2H, m, 5′-H, 9′-H)	128.4, CH	7.54~7.56 (2H, m, 5′-H, 9′-H)	128.3, CH	
2″	/	/	9.202 (s)	150.8, CH	COSY: 8.293 (5″-H)
3″	/	/		126.7, C	
4″	/	/	7.390 (m) in 7.37~7.41 (4H, m)	123.3, CH	HMBC: 153.2 (C-6″)
5″	/	/	8.293 (dd, 8.0, 4.8)	137.1, CH	HMBC: 153.2 (C-6″),
6″	/	/	8.756 (d, 4.8)	153.2, CH	COSY: 7.390, 4″-H (in 7.37~7.41, m), HMBC: 137.1 (C-5″), 150.8 (C-2″), 123.3 (C-4″)
7″	/	/	/	164.7, C	
OH/H_2_O	3.722		4.491, 3.442		

Chemical shifts (δ, in ppm) of ^1^H-NMR/^13^C-NMR for compound **1** were measured at 600/150 MHz in CDCl_3_ with TMS as the internal standard (IS). NMR data of compound **1a** were measured at 400/100 MHz in CDCl_3_. Proton ascription was determined using ^1^H-^1^H COSY as the reference. The multiplicity of carbon was determined according to the broadband decoupling spectra and the DEPT and HMBC spectrum (^1^H → ^13^C).

**Table 2:** Reversal effects of compounds 1 and 1a on P-gp-mediated MDR in HepG2/Dox cells.

Treatment	IC_50_ ± SD ^a^ (µM)		F_res_ ^b^	F_sen_ ^c^
	HepG2	HepG2/Dox		
Doxorubicin	0.17 ± 0.024	6.03 ± 1.15	35.5	1
+ compound **1** (5 µM)	/	2.33 ± 1.37 ***	13.7	2.6
+ compound **1** (10 µM)	/	0.85 ± 0.16 ****	5.0	7.1
+ compound **1a** (5 µM)	/	0.32 ± 0.11 ****	1.9	18.8
+ compound **1a** (10 µM)	/	0.70 ± 0.38 ****	4.1	8.6
+ VRP (10 µM)	/	0.19 ± 0.06 ****	1.1	31.7
Paclitaxel	0.031 ± 0.002	4.03 ± 0.06	130.0	1
+ compound **1** (5 µM)	/	0.12 ± 0.04 ****	3.9	33.6
+ compound **1** (10 µM)	/	0.034 ± 0.002 ****	1.1	118.5
+ compound **1a** (5 µM)	/	0.026 ± 0.004 ****	0.8	155.0
+ compound **1a** (10 µM)	/	0.012 ± 0.006 ****	0.4	335.8
+ VRP (10 µM)	/	0.039 ± 0.005 ****	1.3	103.3
Vinblastine	0.017 ± 0.004	2.38 ± 0.62	140.0	1
+ compound **1** (5 µM)	/	0.026 ± 0.001 ****	1.5	91.5
+ compound **1** (10 µM)	/	0.012 ± 0.002 ****	0.7	198.3
+ compound **1a** (5 µM)	/	0.030 ± 0.0055 ****	1.8	79.3
+ compound **1a** (10 µM)	/	0.017 ± 0.001 ****	1.0	140.0
+ VRP (10 µM)	/	0.036 ± 0.006 ****	2.1	66.1

^a^ Values represent the mean ± SD of three independent experiments performed in triple replicate. ^b^ Fold of resistance (F_res_) to a drug = IC_50_ of drug in HepG2/Dox cells/IC_50_ of drug in HepG2 cells. ^c^ Fold increase in sensitivity (F_sen_) to a drug = IC_50_ of drug alone/IC_50_ of drug with a modulator. *** *p* < 0.001 and **** *p* < 0.0001, compared with the IC_50_ value of a drug alone in HepG2/Dox cells.

**Table 3:** The measured efflux rate, apparent permeability coefficient (×10^−6^ cm·s^−1^), and solution recovery of 1 and 1a on Caco-2 cell monolayers.

Compound	Efflux Ratio	A→B Transport		B→A Transport	
		Papp	Solution Recovery %	Papp	Solution Recovery %
Compound **1**	0.83	9.28 ± 0.88	44.6 ± 2.3	7.71 ± 0.61	50.4 ± 1.7
Compound **1a**	0.89	0.27 ± 0.03	5.3 ± 0.5	0.24 ± 0.02	12.2 ± 0.7
Digoxin	181.00	0.12 ± 0.02	97.9 ± 8.1	22.3 ± 1.02	94.4 ± 1.9

Efflux ratio (ER) = Papp (B→A)/Papp (A→B). Papp: apparent permeability coefficient. For a compound with solution recovery (A→B) ≥ 50%, Papp < 0.6 × 10^−6^ cm · s^−1^ indicates low permeability; Papp ≥ 6.0 × 10^−6^ cm · s^−1^ indicates high permeability; and 0.6 × 10^−6^ cm · s^−1^ ≤ Papp < 6.0 × 10^−6^ cm · s^−1^ indicates medium permeability. An efflux ratio > 2 indicates that it may be a substrate of P-gp; otherwise, it is not.

**Table 4:** SPR analyses of the binding between compound **1** or **1a** to full length human ABCB1.

Receptor	Analyte	K_D_ (M)	Ka (M^−1^s^−1^)	Kd (s^−1^)
Human ABCB1	**1**	5.53 × 10^−6^	6.58 × 10^4^	3.64 × 10^−1^
	**1a**	3.72 × 10^−6^	4.16 × 10^4^	1.55 × 10^−1^

Note: K_D_, equilibrium dissociation constant; Ka, association rate constant; Kd, dissociation rate constant. K_D_ = Kd/Ka.

## Data Availability

The original contributions presented in this study are included in the article; Supplementary Materials. Further inquiries can be directed to the corresponding authors.

## Supplementary Materials

The following supporting information can be downloaded at: https://www.mdpi.com/article/10.3390/ph18081187/s1, Figure S1: Cell survival curve; Figure S2: Spectroscopic data for compound **1**; Figure S3: MS and NMR data for compound **1a**; Figure S4: Method for concentration of compounds **1** and **1a** in Caco-2 cells by LC-MS/MS; Figure S5: Concentration of compounds **1** and **1a** measured in Caco-2 cells.

## Abbreviations

The following abbreviations are used in this manuscript:

CCK-8	Cell counting kit-8
Dox	Doxorubicin
ER	Efflux Ratio
HBSS	Hank’s balanced salt solution
HMBC	Heteronuclear multiple bond correlation
^1^H-^1^H COSY	^1^H-^1^H Correlated spectroscopy
^1^H-NMR	^1^H nuclear magnetic resonance
IC_50_	Half inhibitory concentration
LC-MS/MS	Liquid Chromatography-Tandem Mass Spectrometry
MDR	Multidrug resistance
P-gp	P-glycoprotein
PI	Propidium Iodide
PTX	Paclitaxel
Rh-123	Rhodamine-123
SDS	Sodium dodecyl sulfate
SPR	Surface plasmon resonance
TLC	Thin-Layer Chromatography
VRP	Verapamil
