# Supplementary material for: Polyoxypregnane Aryl Esters Prepared from Metaplexis japonica (Thunb.) Makino and Their Role in Reversing Multidrug Resistance in HepG2/Dox Cells
Source: Pharmaceuticals (Basel). 2025 Aug 12;18(8):1187. doi: 10.3390/ph18081187 (PMC12389735; doi:10.3390/ph18081187)
Supplement: Supplementary file 1 [file pharmaceuticals-18-01187-s001.zip › Figs S1 Cell survival curve + S2 and S3 Spectroscopic data for 1 and 1a.pdf]

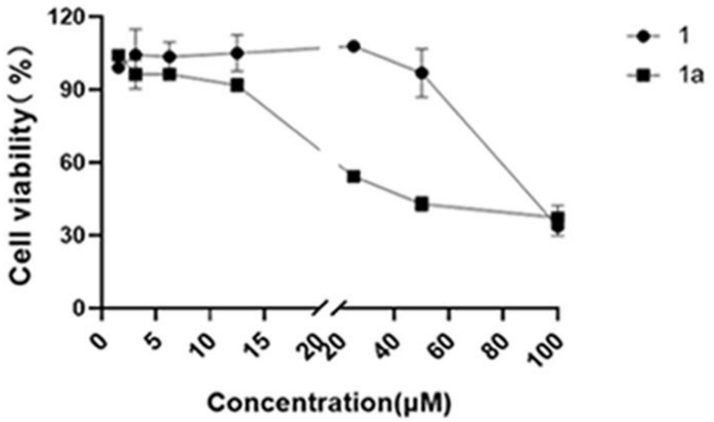

Fig. S1: Cell viability test

A

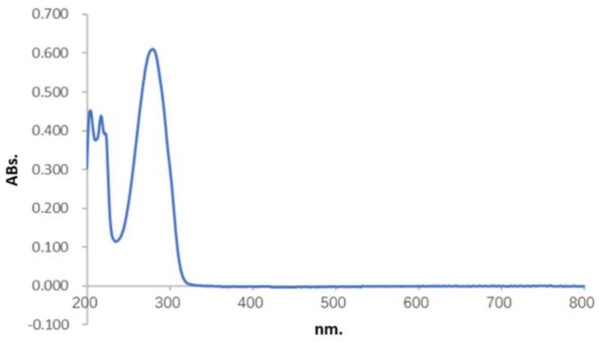

λ（nm）	Absorption Value	log ε
280.0	0.611	2.79
217.0	0.439	2.64
205.0	0.448	2.65

B

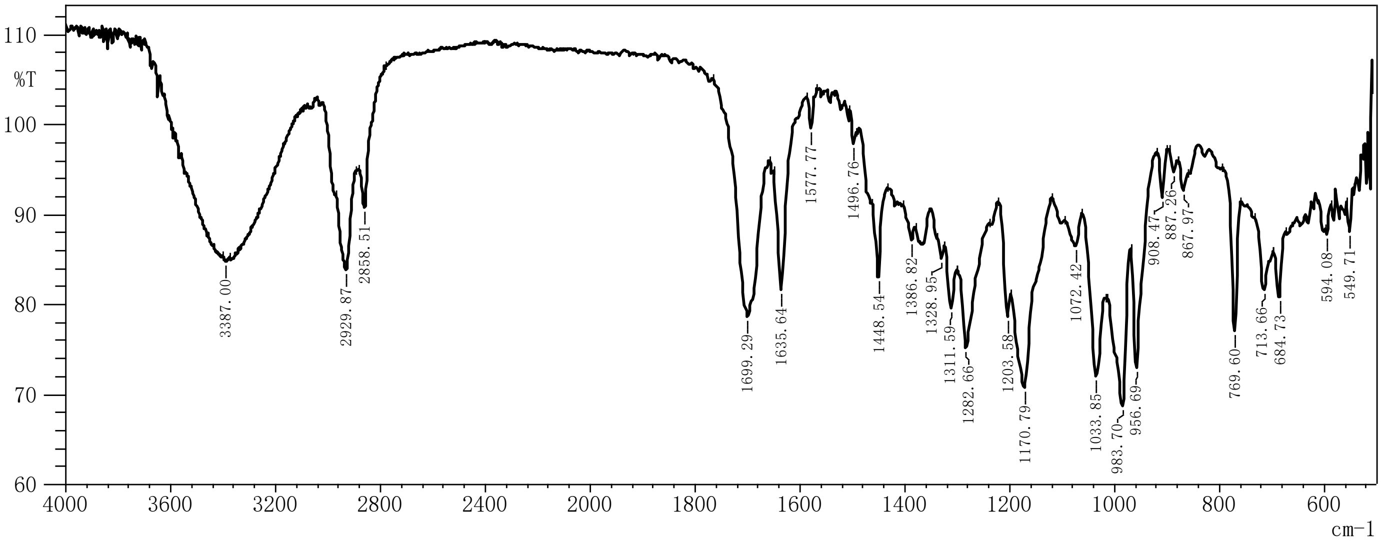

C

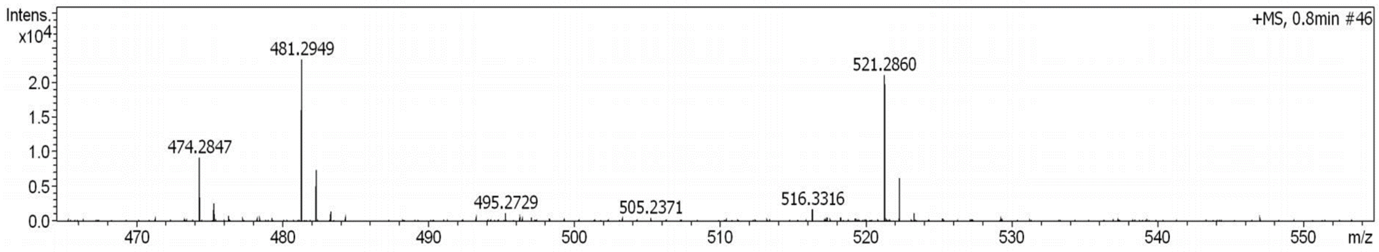

D

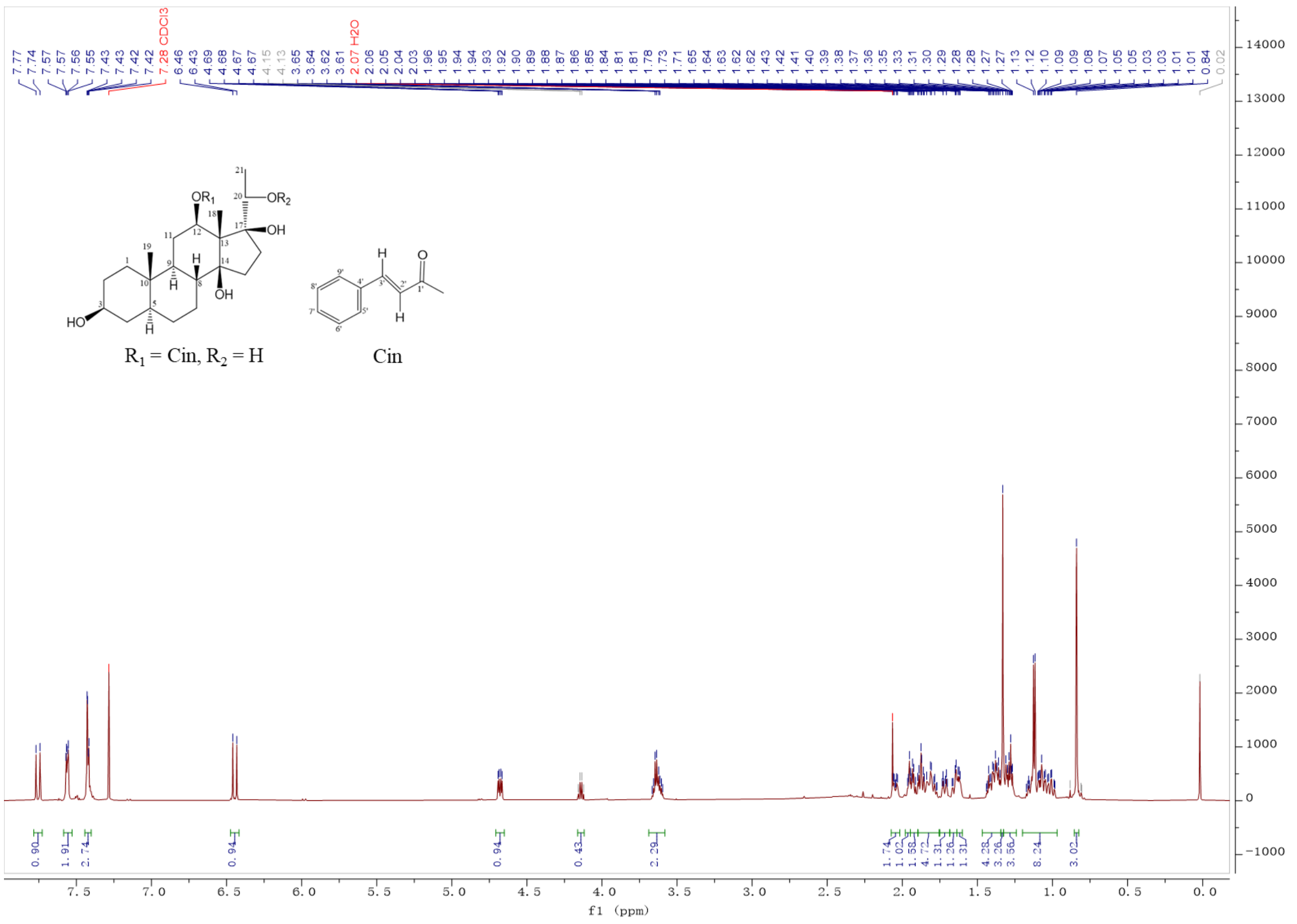

E

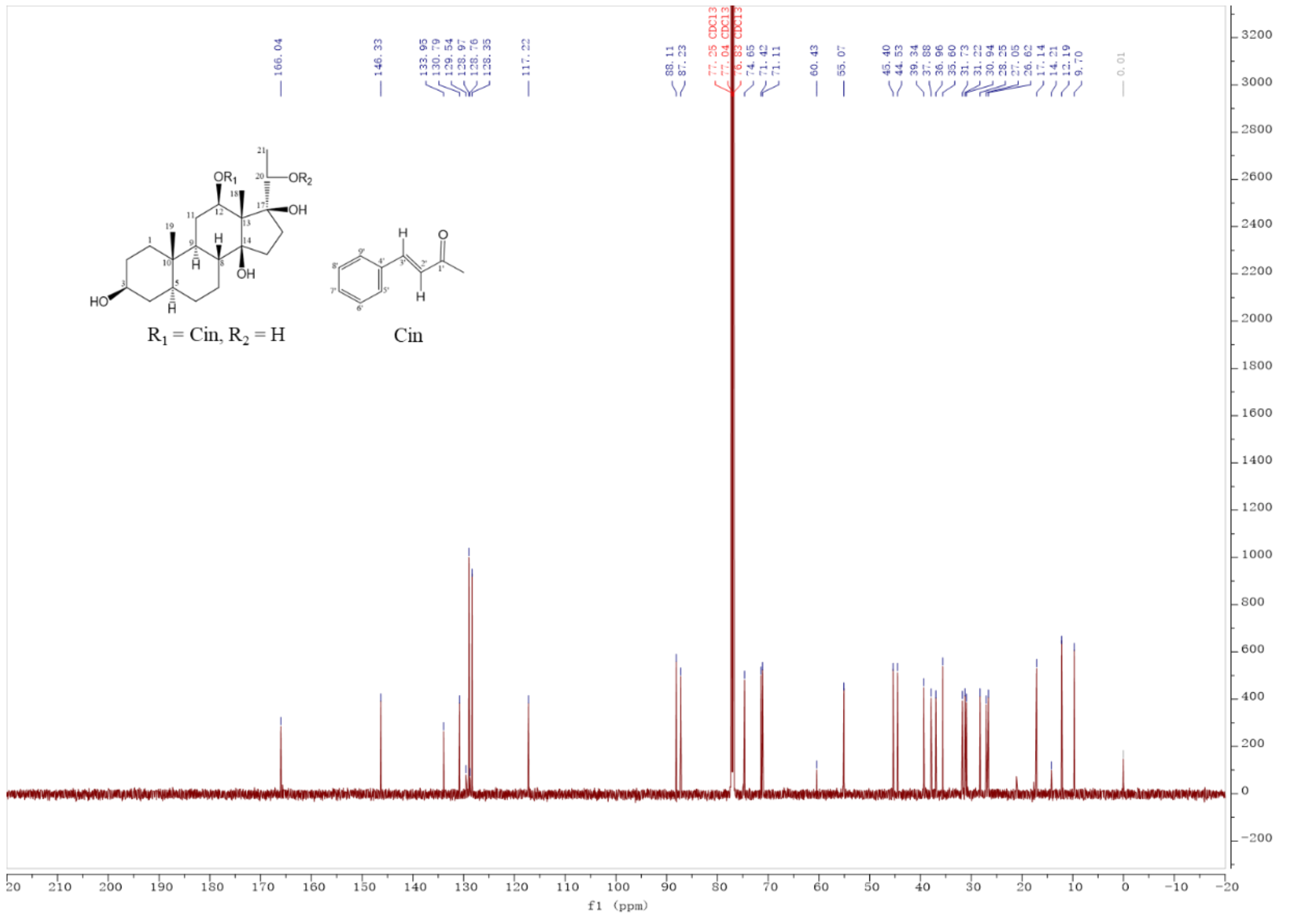

F

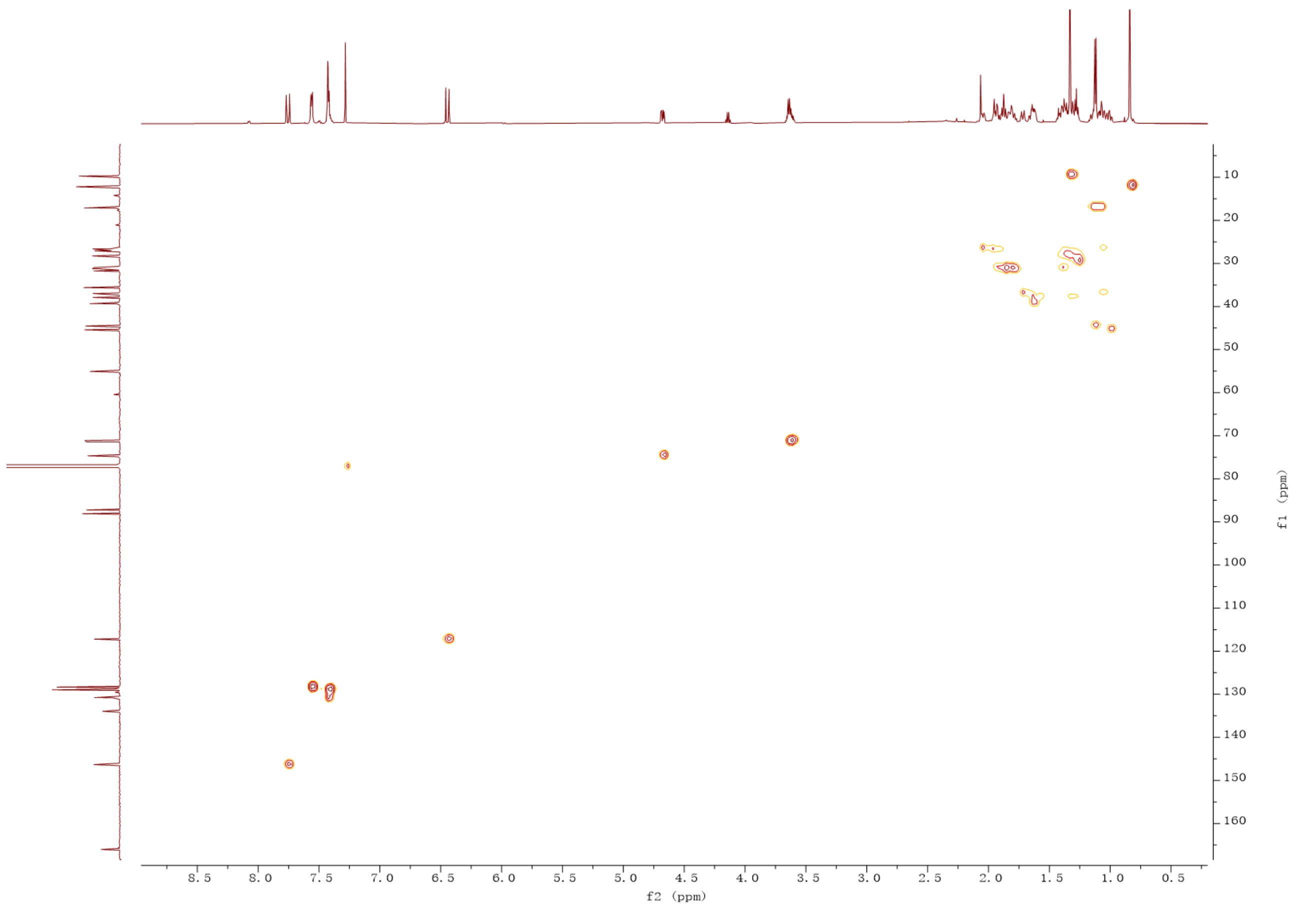

G

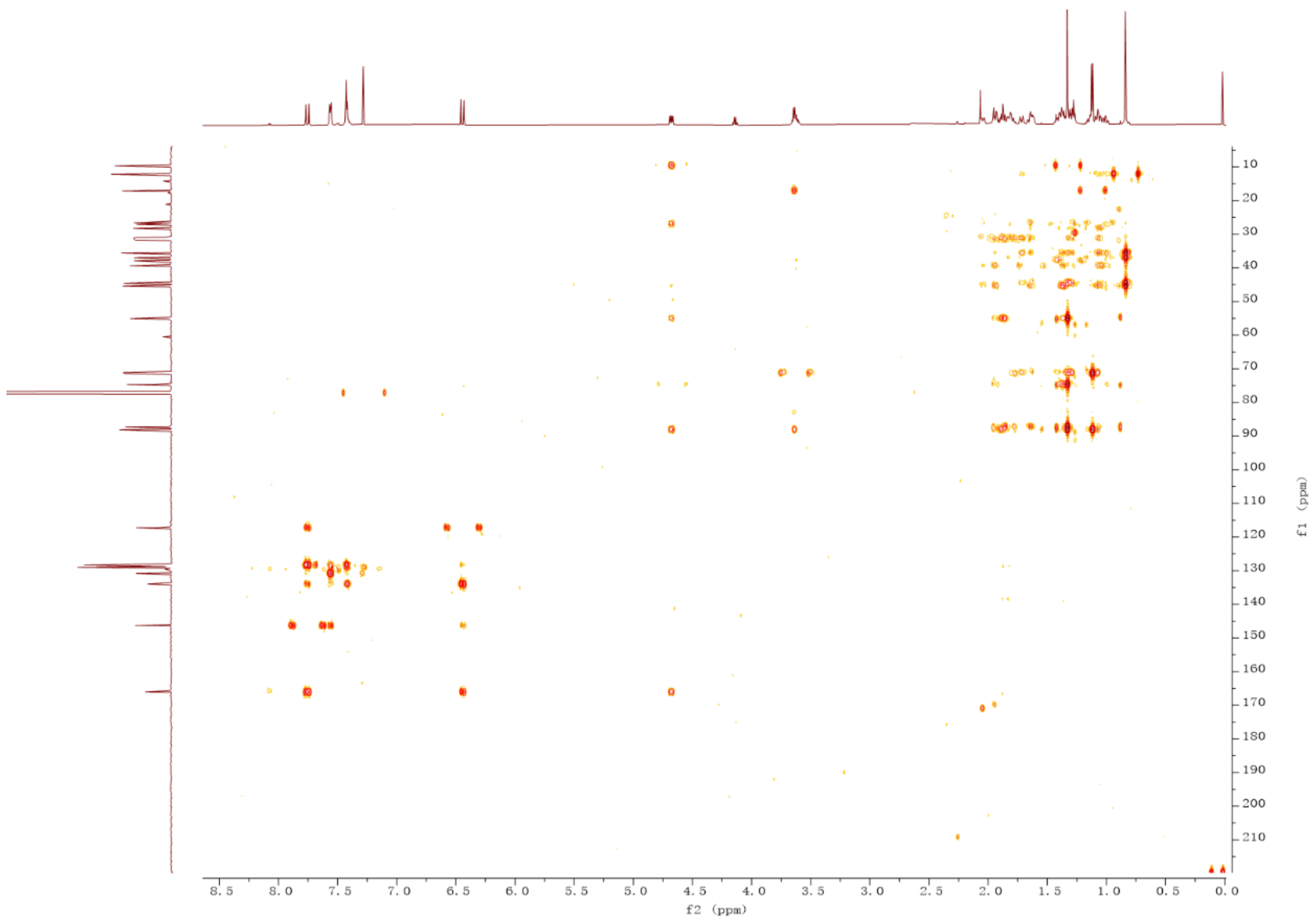

Fig. S2. Spectroscopic data of compound 1: A. UV, B. IR, C. HR-MS, D. ¹H-NMR, E. ¹³C-NMR (BBD), F. ¹³C-NMR (DEPT), G. HMBC.

A

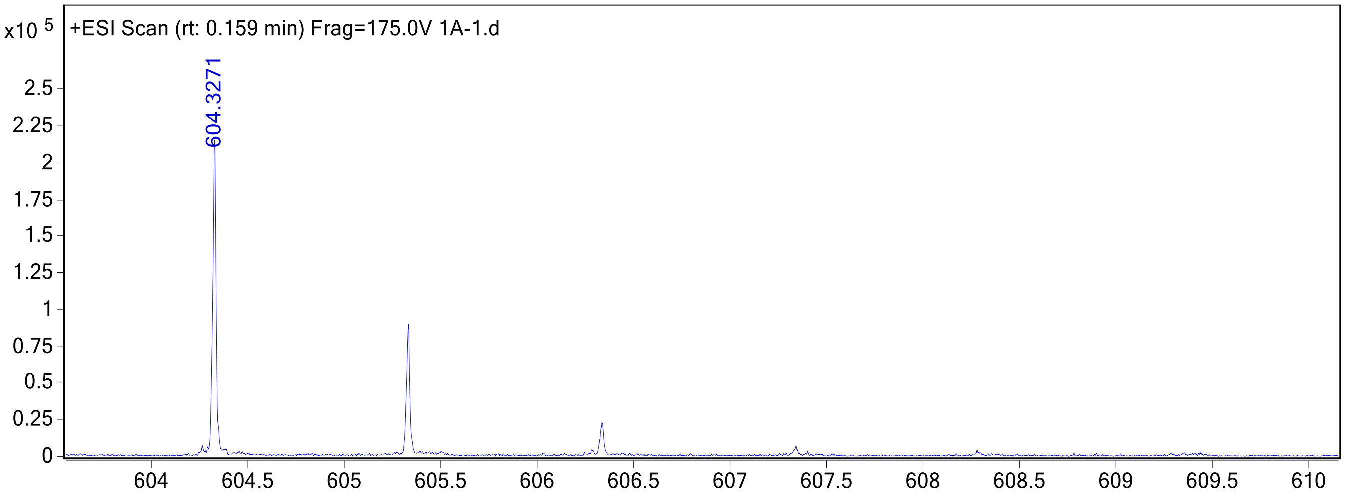

x10 5
+ESI Scan (rt: 0.159 min) Frag=175.0V 1A-1.d
604.3271

B

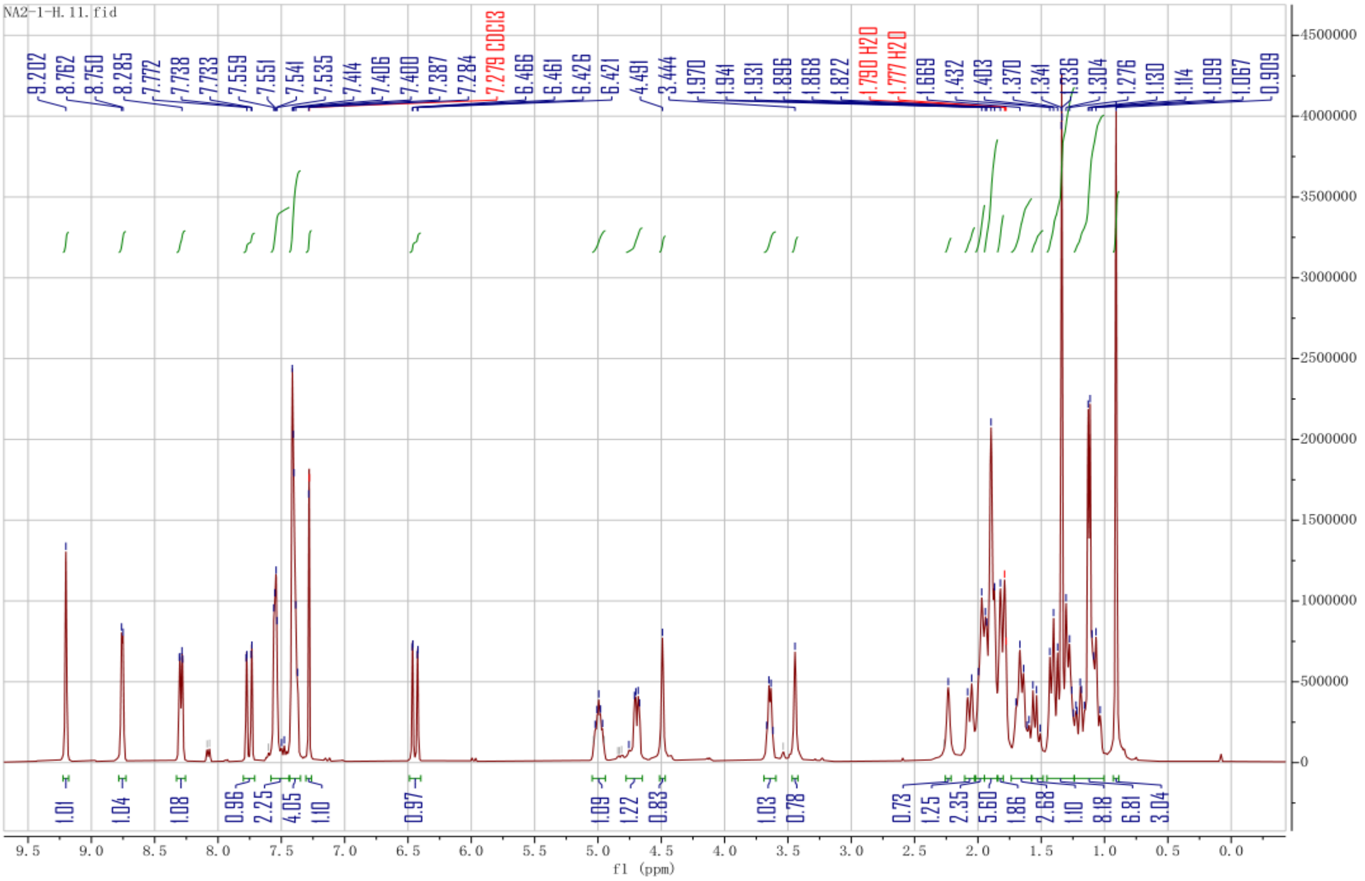

NA2-1-H.11.fid
9.202
8.762
8.750
8.285
7.772
7.738
7.733
7.559
7.551
7.541
7.535
7.414
7.406
7.400
7.387
7.284
7.279 CDCl3
6.466
6.461
6.426
6.421
4.491
3.444
1.970
1.941
1.931
1.896
1.868
1.822
1.790 H2O
1.777 H2O
1.669
1.432
1.403
1.370
1.341
1.336
1.304
1.276
1.130
1.114
1.099
1.067
0.909
1.01
1.04
1.08
0.96
2.25
4.05
1.10
0.97
1.09
1.22
0.83
1.03
0.78
0.73
1.25
2.35
5.60
1.86
2.68
1.10
8.18
6.81
3.04
f1 (ppm)

C

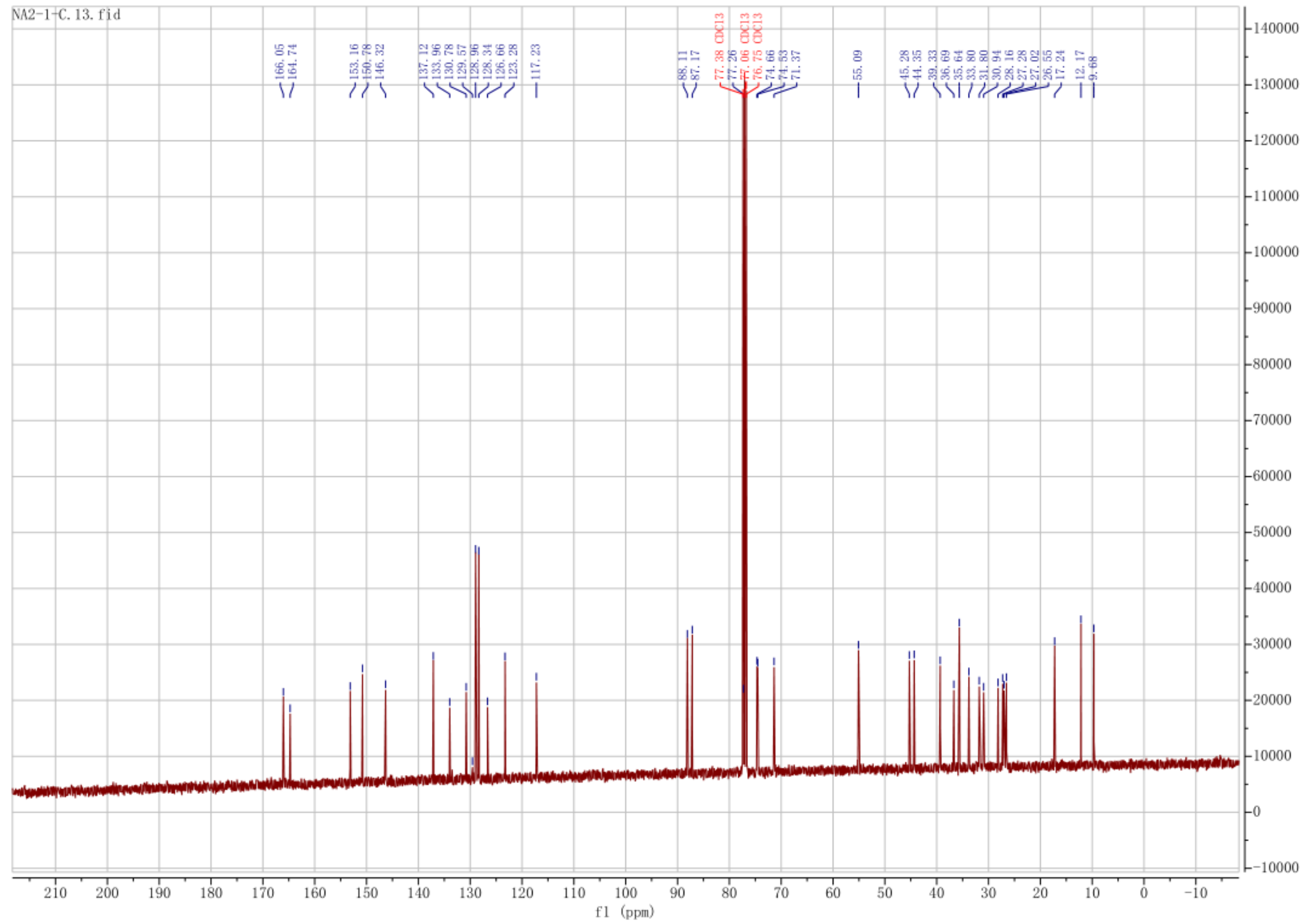
NA2-1-C.13.fid
166.05
164.74
153.16
150.78
146.32
137.12
133.96
130.78
129.57
128.96
128.34
126.66
123.28
117.23
88.11
87.17
77.38 CDCl3
77.26
77.06 CDCl3
76.75 CDCl3
74.66
74.53
71.37
55.09
45.28
44.35
39.33
36.69
35.64
33.80
31.80
30.94
28.16
27.28
27.02
26.55
17.24
12.17
9.68
f1 (ppm)

D

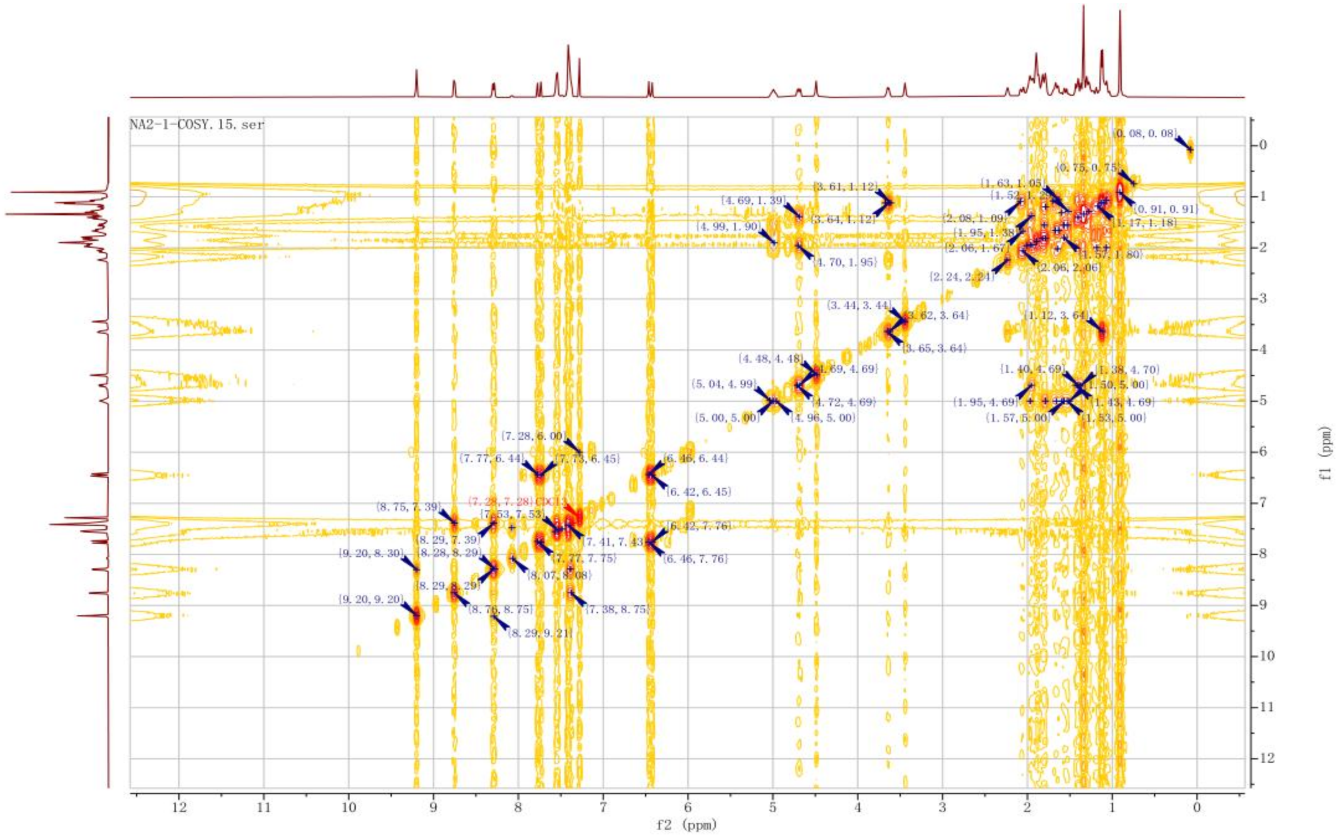
NA2-1-COSY.15.ser
f2 (ppm)
f1 (ppm)

E

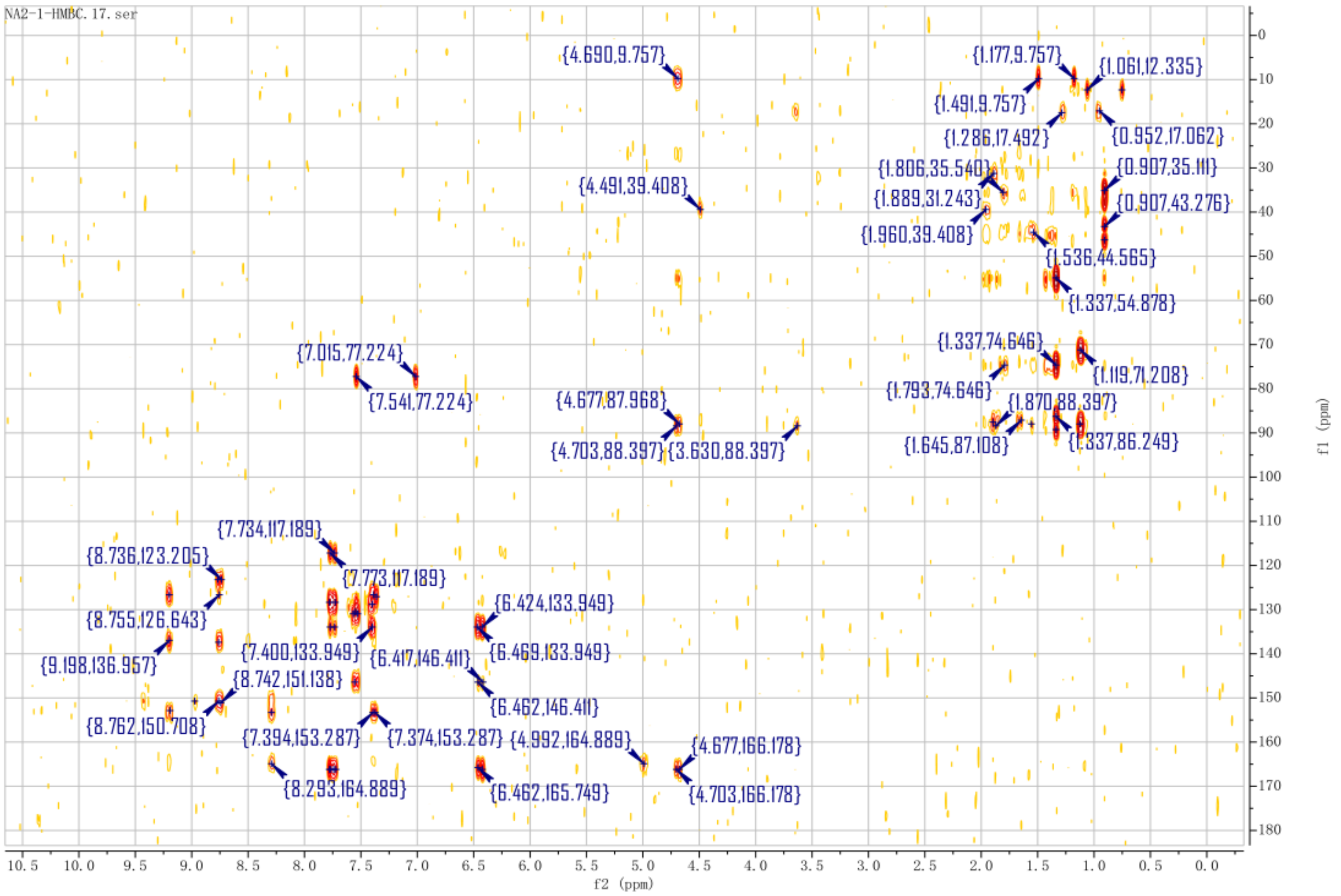

Fig. S3. Spectroscopic data of compound 1a: MS and NMR data of compound 1a. A. HR-MS, B. ^{1}H-NMR, C. ^{13}C-NMR, D. COSY, E. HMBC.
