# Supplementary material for: Polyoxypregnane Aryl Esters Prepared from Metaplexis japonica (Thunb.) Makino and Their Role in Reversing Multidrug Resistance in HepG2/Dox Cells
Source: Pharmaceuticals (Basel). 2025 Aug 12;18(8):1187. doi: 10.3390/ph18081187 (PMC12389735; doi:10.3390/ph18081187)
Supplement: Supplementary file 1 [file pharmaceuticals-18-01187-s001.zip › Figure S4 Method for concentration determination of compounds 1 and 1a in Caco-2 cells by LC-MS.pdf]

Data determination for Caco-2 cell monolayer transport assay

Instruments:

LC: Shimadzu LC-40D X3

MS: SCIEX Triple Quad 6500+

Autosampler: CTC PAL3-RSI

Monitoring ion pairs and mass spectrometry parameters of compounds **1** and **1a** and internal standard (IS)

**Compound**	**MW**	**Formula**	**Ion Transition**		**Polarity**	**DP** (V)	**CE** (V)
			Q1 Ion (*m/z*)	Q3 Ion (*m/z*)			
Tolbutamide (IS)	270.1	$C_{12}H_{18}N_2O_3S$	271.1	155.3	Positive	52	25
A2 (**1**)	498.0	$C_{30}H_{42}O_6$	521.1	373.2	Positive	55	35
2-1 (**1a)**	603.0	$C_{36}H_{45}NO_7$	604.3	124.1	Positive	150	75

Condition for LC

Mobile Phase: A: 0.1% Formic acid in water; B: 0.1% Formic acid in Acetonitrile

Column: Acquity UPLC Ben C18 Column, 1.7 μm, 2.1 × 50 mm

Total Flow: 0.700 mL/min

Mobile phase ratio

Compound	Time (min)	Compositive A (%)	Compositive A (%)
A2 (**1**)	0.00	95	5
	0.40	5	95
	0.80	5	95
	0.81	95	5
	1.00	95	5
2-1 (**1a**)	0.00	95	5
	1.50	5	95
	1.80	5	95
	1.81	95	5
	2.00	95	5

Condition for MS

Ion Source: ESI (Positive ion mode), Scan Type: MRM. Parameters: CUR: 40 psi, IS: 5500 V, TEM: 450°C, GS1: 55 psi, GS2:

60 psi, CAD: 8 psi, EP: 10 V, CXP 11: 11 V.

Concentrations (raw data) of compounds **1** and **1a** measured by this method were included in Supplementary materials S5.
